# Clinical Significance of Peritumoral Adipose Tissue PET/CT Imaging Features for Predicting Axillary Lymph Node Metastasis in Patients with Breast Cancer

**DOI:** 10.3390/jpm11101029

**Published:** 2021-10-15

**Authors:** Jeong Won Lee, Sung Yong Kim, Sun Wook Han, Jong Eun Lee, Sung Hoon Hong, Sang Mi Lee, In Young Jo

**Affiliations:** 1 Department of Nuclear Medicine, College of Medicine, Catholic Kwandong University, International St. Mary’s Hospital, Simgok-ro 100-gil 25, Seo-gu, Incheon 22711, Korea; sads00@naver.com; 2 Department of Surgery, Soonchunhyang University Cheonan Hospital, 31 Suncheonhyang 6-gil, Dongnam-gu, Cheonan 31151, Korea; sykim@schmc.ac.kr (S.Y.K.); chiea@schmc.ac.kr (S.W.H.); elduke00@schmc.ac.kr (J.E.L.); soondaeman00@naver.com (S.H.H.); 3 Department of Nuclear Medicine, Soonchunhyang University Cheonan Hospital, 31 Suncheonhyang 6-gil, Dongnam-gu, Cheonan 31151, Korea; 4 Department of Radiation Oncology, Soonchunhyang University Cheonan Hospital, 31 Suncheonhyang 6-gil, Dongnam-gu, Cheonan 31151, Korea

**Keywords:** breast cancer, F-18 fluorodeoxyglucose, lymph node metastasis, PET/CT, texture analysis

## Abstract

We investigated whether textural parameters of peritumoral breast adipose tissue (AT) based on F-18 fluorodeoxyglucose (FDG) PET/CT could predict axillary lymph node metastasis in patients with breast cancer. A total of 326 breast cancer patients with preoperative FDG PET/CT were retrospectively enrolled. PET/CT images were visually assessed and the maximum FDG uptake of axillary lymph nodes (LN SUVmax) was measured. From peritumoral breast AT, 38 textural features of PET imaging were extracted. The diagnostic ability of PET based on visual analysis, LN SUVmax, and textural features of peritumoral breast AT for predicting axillary lymph node metastasis were assessed using the area under the receiver operating characteristic curve (AUC) values. Among the 38 peritumoral breast AT textural features, grey-level co-occurrence matrix (GLCM) entropy showed the highest AUC value (0.830) for predicting axillary lymph node metastasis. The value of GLCM entropy was higher than that of visual analysis (0.739; *p* < 0.05) and the AUC value was comparable to that of LN SUVmax (0.793; *p* > 0.05). In the subgroup analysis of patients with negative findings on visual analysis, GLCM entropy still showed a high diagnostic ability (AUC: 0.759) in predicting lymph node metastasis. The findings suggest a potential diagnostic role of PET/CT imaging features of peritumoral breast AT in predicting axillary lymph node metastasis in patients with breast cancer.

## 1. Introduction

Breast cancer is the primary cause of the incidence of and death from female cancer globally [1]. In patients with breast cancer, axillary lymph node metastasis is one of the most significant clinical factors dictating the treatment strategy and predicting survival [2,3]. Axillary lymph node dissection is recommended for breast cancer patients involving clinically and biopsy-proven positive axillary lymph nodes [2]. Meanwhile, sentinel lymph node biopsy is suggested as the gold standard for axillary lymph node staging of patients with clinically negative or suspicious lymph nodes but negative biopsy results [2,4]. However, sentinel lymph node biopsy showed a 9.8% false-negative rate in a previous clinical trial and, despite a lower morbidity than that of axillary lymph node dissection, surgical complications such as lymphedema were reported in 3.7–6.9% of patients undergoing sentinel lymph node sampling [5,6,7]. Therefore, a noninvasive diagnostic method is highly desirable for accurate axillary lymph node staging [4,8]. F-18 fluorodeoxyglucose (FDG) positron emission tomography/computed tomography (PET/CT) is notable for its exquisite diagnostic ability for detecting metastasis in various malignant diseases; therefore, it is one of the imaging examinations investigated for axillary lymph node staging [4,8,9,10]. Unfortunately, in a recent meta-analysis study, FDG PET/CT showed a sensitivity of only 52% for detecting axillary lymph node metastasis, whereas the specificity was 92%, suggesting the limited use of standalone FDG PET/CT in axillary nodal staging [9]. Hence, a number of studies have attempted to develop diverse methods to improve the diagnostic accuracy of FDG PET/CT [4,11].

Recently, increasing evidence has been suggesting that breast cancer cells interact substantially with adipocytes in the vicinity of cancer cells [12,13]. Breast cancer cells induce significant phenotypic and functional alterations with metabolic reprogramming of peritumoral adipocytes [12,14]. Altered adipocytes promote the growth, invasion, and metastasis of breast cancer cells and induce an inflammatory microenvironment in the peritumoral adipose tissue (AT) [12,13]. Previous studies reported that the adipocyte alterations in peritumoral breast AT can be evaluated via imaging features of CT and magnetic resonance imaging (MRI) [15,16,17]. Further, these imaging features of peritumoral breast AT based on CT and MRI were significantly associated with axillary lymph node metastasis [15,16,17]. The interaction between breast cancer cells and AT leads to enhanced glycolysis of adipocytes and inflammation in peritumoral AT [12,14], suggesting that peritumoral breast AT might also exhibit distinct FDG PET/CT features in patients with breast cancer, which could also have a significant association with tumor invasiveness and metastasis. In previous studies, FDG uptake of peritumoral AT in pancreatic, gastric, and prostate cancers demonstrated a significant association with tumor aggressiveness and prognosis [18,19,20]. However, until now, the clinical significance of FDG PET/CT imaging features of peritumoral breast AT have yet to be reported.

In this study, we measured the textural features of peritumoral breast AT on FDG PET/CT and investigated their potential diagnostic role in predicting axillary lymph node metastasis in patients with breast cancer.

## 2. Materials and Methods

### 2.1. Study Population

**Figure 1:**
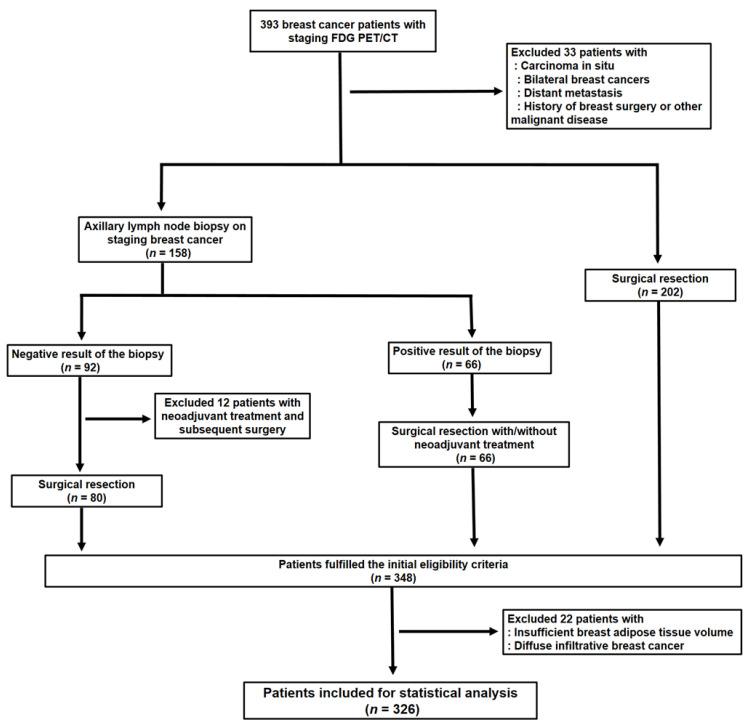
Flow diagram showing the process of patient enrollment.

Electronic medical records were retrospectively reviewed for 393 female patients with histopathologically confirmed invasive breast cancer who underwent FDG PET/CT for staging workup between February 2012 and December 2016 at the Soonchunhyang University Cheonan Hospital. Of the 393 patients, we excluded 67 patients (1) who were diagnosed with carcinoma in situ, (2) who had bilateral breast cancer lesions, (3) who had distant metastatic lesions based on staging workup, (4) who had a previous history of breast surgery or other malignant diseases, (5) who had a negative result of staging axillary lymph node biopsy and received neoadjuvant treatment before surgical resection which prevented histopathological confirmation of axillary lymph nodes, (6) who had insufficient peritumoral breast AT volume, and (7) who had diffuse infiltrative breast cancer (diffuse, infiltrative, and non-mass enhancement features on MRI with diffusely increased FDG uptake in the PET/CT images). Therefore, 326 patients were finally enrolled in the study, comprised of (1) the patients who underwent surgical resection of breast cancer without axillary lymph node biopsy on staging workup, (2) the patients who had positive results of staging axillary lymph node biopsy followed by surgical resection with or without neoadjuvant treatment, and (3) the patients who had negative results of staging axillary lymph node biopsy further confirmed via subsequent surgical resection without neoadjuvant treatment (Figure 1).

For the 158 patients with axillary lymph node biopsy on staging workup, ultrasonography-guided axillary lymph node biopsy was performed for lymph nodes with the most intense FDG uptake or the largest lymph node. The initial treatment was performed within 14 days (median, 5 days) after FDG PET/CT in all the enrolled patients. All the enrolled patients underwent breast-conserving surgery or total mastectomy with or without neoadjuvant treatment after staging workup. Sentinel lymph node biopsy using blue dye or axillary lymph node dissection was performed in all the patients irrespective of preoperative lymph node biopsy and neoadjuvant treatment. Axillary lymph node metastasis was diagnosed based on both the frozen-section findings and the standard histopathological results. Based on the histopathological investigations and clinical conditions, the patients underwent adjuvant treatment including chemotherapy, radiotherapy, and/or hormone therapy after the surgery. All the clinical and histopathological data of the 326 patients were retrieved from electronic medical records.

### 2.2. FDG PET/CT Scans 

FDG PET/CT scans were performed using a Biograph mCT 128 scanner (Siemens Healthcare, Knoxville, TN, USA). Before PET/CT scanning, the patients were required to fast for at least 6 h to ensure a blood glucose level < 150 mg/dL. Sixty minutes after intravenous injection of approximately 4.07 MBq/kg of FDG, we performed an FDG PET/CT scan from the skull base to the upper thigh in the supine position. A CT scan was performed at 100 mA and 120 kVp of automated dose modulation and slice thickness of 5 mm without intravenous contrast agent injection. A PET scan was performed with 90 s in each bed position using the three-dimensional acquisition mode. PET images were reconstructed using the point spread function based on the Gauss and Allpass filter algorithm and time-of-flight reconstruction (21 subsets and two iterations) on a 128 × 128 matrix with attenuation correction [21,22].

### 2.3. FDG PET/CT Image Analysis

**Figure 2:**
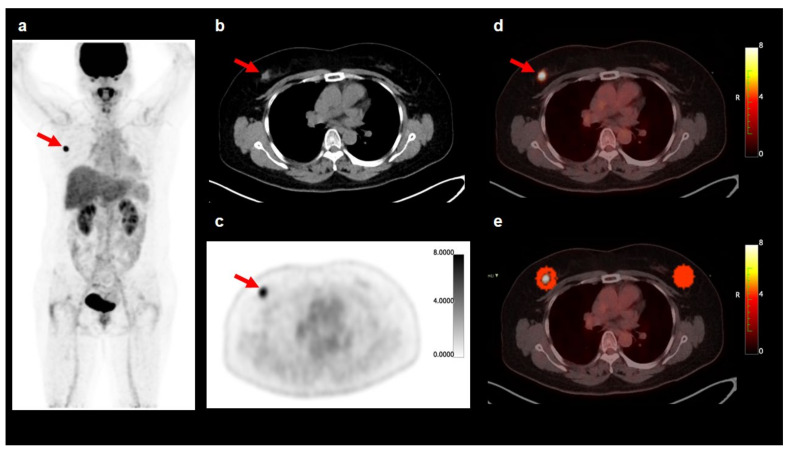
Maximal intensity projection image (**a**), transaxial CT image (**b**), transaxial PET image (**c**), and fused transaxial images (**d**,**e**) of F-18 fluorodeoxyglucose (FDG) PET/CT illustrating volumes of interest (VOIs) for measuring breast adipose tissue imaging features. A 53-year-old woman underwent preoperative FDG PET/CT for right breast cancer which was histopathologically diagnosed with invasive ductal carcinoma. The FDG PET/CT images revealed a breast cancer lesion showing increased FDG uptake with the maximum standardized uptake value of 9.39 (arrows in (**a**–**d**)). A spheroid-shaped VOI that covers the peritumoral breast tissue within a 1 cm distance to the margin of the breast cancer lesion was manually drawn, and another VOI of the same size was drawn in the same quadrant of the contralateral breast tissue. Within the VOIs in the bilateral breast tissues, the area with CT attenuation ranging between −200 and −50 Hounsfield units was automatically delineated and defined as the area of the peritumoral and contralateral breast tissue (**e**).

The PET/CT images were retrospectively analyzed by two physicians specializing in nuclear medicine blinded to the clinical and histopathological results. First, the PET/CT images were visually assessed and all the patients were categorized into the patients with a negative or positive axillary lymph node lesion based on the results of visual analysis. Axillary lymph nodes with increased FDG uptake exceeding the background uptake were interpreted as positive findings. Discrepancies between the two readers were resolved by consensus. Subsequently, a quantitative analysis of the FDG PET/CT images was performed using open-source LIFEx software version 7.0.0 (www.lifexsoft.org; accessed 25 May 2021) [23]. A spheroid-shaped volume of interest (VOI) was manually drawn around the primary breast cancer lesion and the maximum standardized uptake value (SUV) of the primary tumor was measured in all the patients. The maximum SUV of axillary lymph nodes (LN SUVmax) was also measured in the patients who had ipsilateral axillary lymph nodes in the PET/CT images. AT within 1 cm from the tumor margin was defined as peritumoral breast AT [15]. A VOI was manually drawn to include the entire breast cancer lesion and the surrounding breast tissue within a 1 cm distance to the tumor margin, and subsequently, another VOI of the same size was manually drawn over the contralateral breast tissue in the same quadrant (Figure 2) [15]. Areas of CT attenuation ranging between −200 Hounsfield units (HU) and −50 HU within VOIs in the ipsilateral and contralateral breast tissues was defined as peritumoral and contralateral breast AT, respectively [15]. Before the extraction of textural parameters, all the VOIs were manually scrutinized to avoid the spillover FDG activity of the cancer lesion. A total of 38 textural features of the PET images were extracted from each area of peritumoral and contralateral breast AT, including seven first-order features and 31 higher-order features comprising six grey-level co-occurrence matrix (GLCM) features, three neighborhood grey-level different matrix (NGLDM) features, 11 grey-level run-length matrix (GLRLM) features, and 11 grey-level zone-length matrix (GLZLM) features (Table S1) [23,24,25]. 

### 2.4. Statistical Analysis

**Figure 3:**
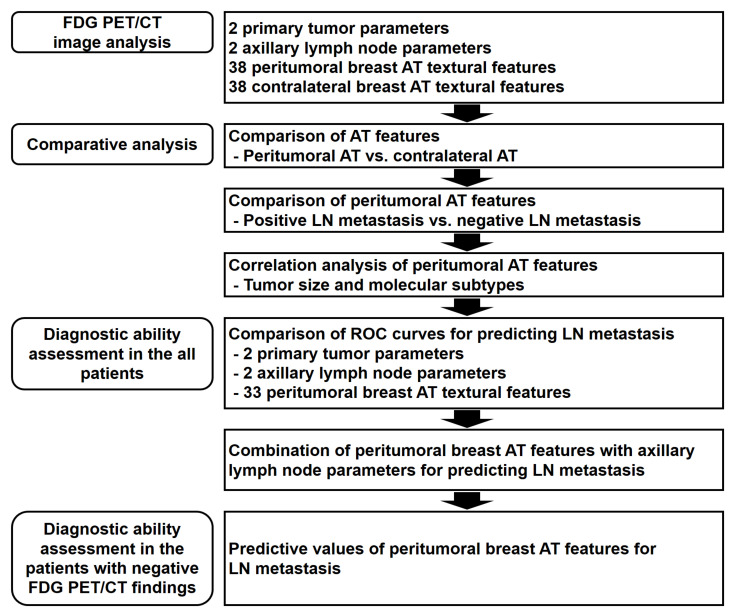
Schematic overview of the workflow. AT, adipose tissue; LN, lymph node; ROC, receiver operating characteristic.

Schematic presentation of the overall workflow in this study is shown in Figure 3. Paired *t*-test was performed to evaluate the differences in textural features between peritumoral and contralateral breast AT. A Student’s *t*-test was performed to compare the differences in peritumoral breast AT textural features between the patients with and without axillary lymph node metastasis. The differences in textural features according to the molecular subtypes and results of visual analysis of the PET/CT images were assessed via the Kruskal–Wallis test with Dunn’s test for post-hoc comparisons. Spearman’s rank correlation was performed to evaluate the relationship between tumor size and textural features. A chi-squared test was performed to compare the distribution of patients with axillary lymph node metastasis according to the molecular subtypes. The diagnostic abilities of visual analysis, LN SUVmax, primary breast cancer parameters, and peritumoral breast AT textural features were evaluated based on the area under the receiver operating characteristic (ROC) curve (AUC) values. The optimal cutoff values of the parameters were identified using the Youden index [26]. Using the optimal cutoff values, the sensitivity, specificity, positive predictive value (PPV), and negative predictive value (NPV) of the parameters for predicting axillary lymph node metastasis were assessed. The differences in the AUC between the parameters were compared using DeLong’s test after applying the Bonferroni adjustment for multiple comparisons [27]. MedCalc Statistical Software version 20.009 (MedCalc Software Ltd., Ostend, Belgium) was used for all the statistical analyses. A *p*-value < 0.05 was regarded as statistically significant.

## 3. Results

### 3.1. Patient Characteristics

The clinical characteristics of the 326 female patients with breast cancer are shown in Table 1. Of the enrolled patients, 119 patients (36.5%) were histopathologically diagnosed with axillary lymph node metastasis. All the patients with lymph node metastasis carried axillary lymph node metastasis. Neoadjuvant chemotherapy was performed in 30 patients (9.2%) who manifested axillary lymph node metastasis confirmed histopathologically with lymph node biopsy before the start of neoadjuvant treatment. Sentinel lymph node biopsy was performed in 184 patients (56.4%), axillary lymph node dissection—in the remaining 142 patients (43.6%). Among all the patients, measurable axillary lymph nodes were observed in the PET/CT images of 278 patients (85.3%), and therefore, LN SUVmax was measured in all these patients. Of them, 116 patients (41.7%; 116 out of the 278 patients) manifested axillary lymph node metastasis. 

The patients with triple-negative cancer (20/38; 52.6%) and human epidermal growth factor receptor 2 (HER2)-enriched cancer (18/42; 42.9%) showed higher proportions of patients with axillary lymph node metastasis than those with luminal A cancer (19/64; 29.7%) and luminal B cancer (62/182; 34.1%), but only borderline statistical significance was shown (*p* = 0.081). 

### 3.2. Comparison of Breast AT Imaging Features

To evaluate the differences in the imaging characteristics of peritumoral breast AT and contralateral breast AT, 38 textural features of peritumoral breast AT were compared pairwise with those of the contralateral side (Table S2). Based on the pairwise comparison analysis, except for GLRLM run-length nonuniformity, 37 textural features showed significantly different values between peritumoral and contralateral breast AT (*p* = 0.005 for NGLDM busyness and *p* < 0.001 for all the other 36 textural features). 

Subsequently, we compared 38 peritumoral breast AT textural features in the 119 patients with axillary lymph node metastasis and in the 207 patients without axillary lymph node metastasis to evaluate the relationship between peritumoral breast AT imaging features and axillary lymph node metastasis (Table S3). Among the 38 textural features, significant differences of values were observed in 33 textural features between the patients with and without axillary lymph node metastasis (*p* < 0.05).

The relationship of textural features of peritumoral breast AT with tumor size and molecular subtypes of breast cancer was also assessed. The size of the primary tumor revealed weak-to-moderate correlations with 33 peritumoral breast AT textural features (Table S4; *p* < 0.05; correlation coefficient, −0.434–0.549). Correlation analysis for peritumoral breast AT textural features and molecular subtypes (Table S5) showed significantly different values of 31 textural features according to the molecular subtypes of breast cancers (*p* < 0.05). On post hoc analysis, the patients with HER2-enriched and/or triple-negative breast cancers showed significantly different values of textural features from those with luminal A and/or luminal B breast cancers. 

### 3.3. Diagnostic Ability for Predicting Axillary Lymph Node Metastasis

The diagnostic ability of visual analysis, LN SUVmax, size and the maximum SUV of the primary tumor, and textural features of peritumoral breast AT for predicting axillary lymph node metastasis were assessed (Table 2 and Table S6). Among the 38 textural features of peritumoral breast AT, 33 textural parameters that showed significant differences between the patients with and without axillary lymph node metastasis were included in the assessment. On the visual analysis of PET/CT, positive findings were detected in 77 patients (23.6%), and the remaining 249 patients (76.4%) were considered negative. Of the 77 patients with positive PET/CT findings, 64 patients were histopathologically diagnosed with axillary lymph node metastasis. Of the 249 patients with negative PET/CT findings, 55 patients were histopathologically diagnosed with axillary lymph node metastasis. Sensitivity, specificity, PPV, and NPV of the visual analysis of PET/CT were 53.8%, 93.7%, 83.1%, and 77.9%, respectively, with an AUC value of 0.739 (95% confidence interval, 0.689–0.785). For the 278 patients with measurable axillary lymph nodes in the PET/CT images, LN SUVmax showed a sensitivity of 56.9%, a specificity of 91.4%, a PPV of 82.5%, and an NPV of 74.7% for diagnosing axillary lymph node metastasis using the optimal cutoff value of 1.58 with an AUC value of 0.793 (95% CI, 0.741–0.839).

**Figure 4:**
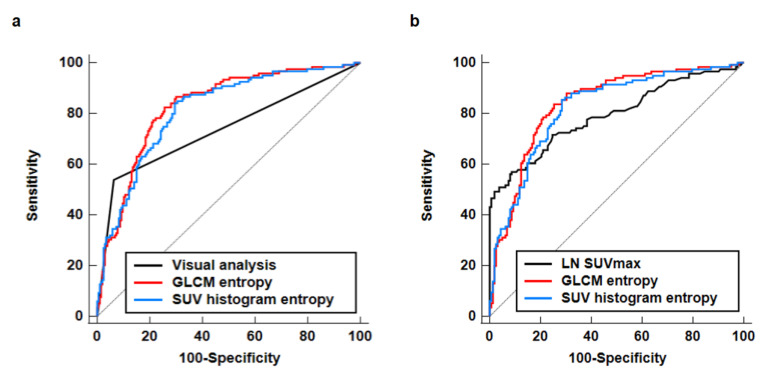
Comparison of the receiver operating characteristic curves for grey-level co-occurrence matrix entropy (GLCM entropy) and standardized uptake value histogram-based entropy (SUV histogram entropy) of peritumoral breast adipose tissue and visual analysis of PET/CT (**a**). Comparison of the receiver operating characteristic curves for GLCM entropy and SUV histogram entropy of peritumoral breast adipose tissue and the maximum SUV of the axillary lymph node (LN SUVmax) (**b**).

Among the 33 textural parameters of peritumoral breast AT, GLCM entropy (the randomness of grey-level voxel pairs) and SUV histogram entropy (the randomness of the distribution in the SUV histogram) showed a high diagnostic ability for predicting axillary lymph node metastasis, demonstrating an AUC value > 0.800 (0.830 (95% CI, 0.784–0.869) for GLCM entropy and 0.815 (95% CI, 0.768–0.855) for SUV histogram entropy). Both GLCM entropy and SUV histogram entropy showed high sensitivity (82.4% for GLCM entropy and 84.0% for SUV histogram entropy) and high NPV (88.0% for GLCM entropy and 88.5% for SUV histogram entropy) for predicting axillary lymph node metastasis. A comparison of ROC curves revealed significantly higher AUC values of GLCM entropy and SUV histogram entropy than the results of visual analysis after Bonferroni correction (*p* = 0.006 for GLCM entropy and *p* = 0.025 for SUV histogram entropy; Figure 4a). Meanwhile, no significant differences in the AUC values were found between both peritumoral breast AT features and LN SUVmax (*p* = 0.567 for GLCM entropy and *p* = 0.804 for SUV histogram entropy; Figure 4b). In addition to GLCM entropy and SUV histogram entropy, GLCM contrast (0.780), GLZLM zone-length nonuniformity (0.766), GLZLM high grey-level zone emphasis (0.765), GLRLM high grey-level run emphasis (0.763), and GLRLM short-run high grey-level emphasis (0.761) showed an AUC value > 0.750 (Table 2). 

The predictive value for axillary lymph node metastasis was further enhanced by combining axillary lymph node findings and textural features of peritumoral breast AT with AUC values >0.800 (GLCM entropy and SUV histogram entropy) (Table 3). On the combination of GLCM entropy and axillary lymph node findings, the prevalence of axillary lymph node metastasis was 95.1–95.2% in the patients with GLCM entropy >3.16 and positive axillary lymph node findings on PET/CT (positive on visual analysis or LN SUVmax > 1.58). Meanwhile, among the patients with GLCM entropy ≤ 3.16 and negative findings (negative on visual analysis or LN SUVmax ≤ 1.58), only 9.5–10.7% of the patients were diagnosed with axillary lymph node metastasis. Similarly, on the combination of SUV histogram entropy and axillary lymph node findings, 93.8–95.5% of the patients with SUV histogram entropy >1.90 and positive axillary lymph node findings had axillary lymph node metastasis, whereas 10.5–11.9% of the patients with ≤1.90 and negative axillary lymph node findings were diagnosed with axillary lymph node metastasis.

### 3.4. Predictive Value of GLCM Entropy and SUV Histogram Entropy in Patients with Negative Axillary Lymph Node Findings 

**Figure 5:**
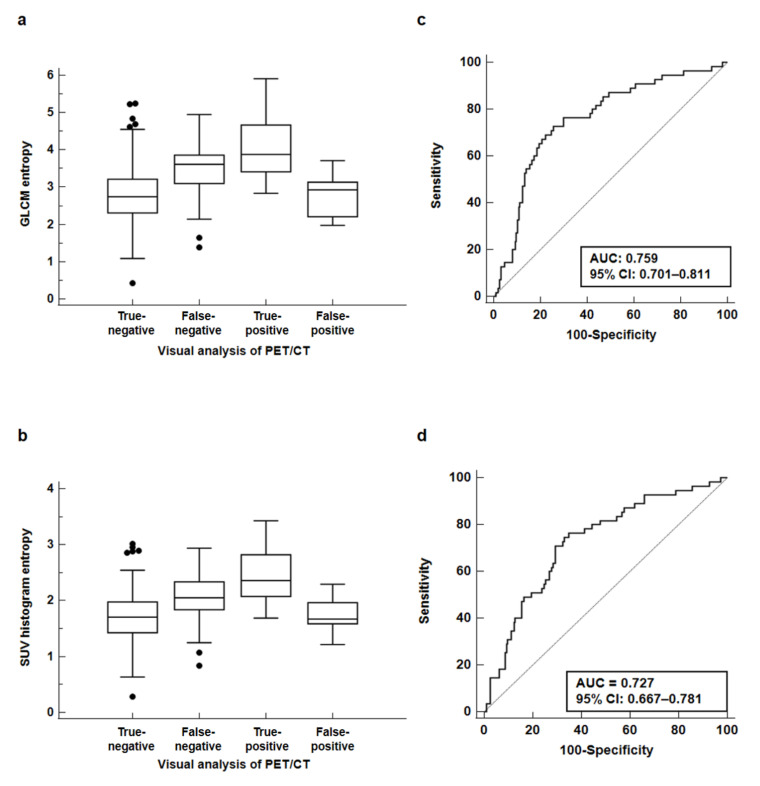
Distribution of the grey-level co-occurrence matrix entropy (GLCM entropy) (**a**) and the standardized uptake value (SUV) histogram entropy values (**b**) based on the results of the visual analysis of PET/CT. The receiver operating characteristic curves for GLCM entropy (**c**) and SUV histogram entropy (**d**) for predicting patients with axillary lymph node metastasis among the patients with negative findings on the visual analysis of PET/CT.

We classified all the enrolled patients into four subgroups based on the results of visual analysis of PET/CT: 194 patients with true-negative findings, 55 patients with false-negative findings, 64 patients with true-positive findings, and 13 patients with false-positive findings. On the Kruskal–Wallis test, there were significant differences in GLCM entropy and SUV histogram entropy between the four groups (*p* < 0.001; Figure 5a,b). Post hoc analysis using Dunn’s test revealed that the patients with true-positive findings had the highest GLCM entropy (4.05 ± 0.76) and SUV histogram entropy (2.45 ± 0.44), showing significantly higher values than all the other three subgroups (*p* < 0.05 for all). The patients with false-negative readings also showed significantly higher values of both GLCM entropy (3.48 ± 0.72) and SUV histogram entropy (2.06 ± 0.44) than those with true-negative (2.79 ± 0.78 for GLCM entropy and 1.71 ± 0.45 for SUV histogram entropy) and false-positive (2.79 ± 0.55 for GLCM entropy and 1.74 ± 0.28 for SUV histogram entropy) results (*p* < 0.05 for all), whereas no significant difference in GLCM entropy and SUV histogram entropy was found between the patients with true-negative and false-positive findings (*p* > 0.05 for all). 

On the basis of the comparative analysis results, we further evaluated whether GLCM entropy and SUV histogram entropy can be used to predict axillary lymph node metastasis among patients with negative findings on the visual analysis of PET/CT images. Thus, GLCM entropy and SUV histogram entropy can be used to distinguish patients with false-negative findings from patients with true-negative findings. In the ROC curve analysis, GLCM entropy showed a high diagnostic ability for predicting axillary lymph node metastasis among the patients with negative findings of the visual analysis of PET/CT, showing an AUC value of 0.759 (95% CI, 0.701–0.811) (Figure 5c). Using the optimal cutoff value of 2.83, GLCM entropy showed a sensitivity of 81.8%, a specificity of 56.2%, a PPV of 34.6%, and an NPV of 91.6%. In addition, the AUV of SUV histogram entropy was 0.727 (95% CI, 0.667–0.781), demonstrating a sensitivity of 81.8%, a specificity of 52.1%, a PPV of 32.6%, and an NPV of 91.0%, using the cutoff value of 1.72 (Figure 5d). 

## 4. Discussion

Recently, a number of studies has revealed that the interrelationship between breast cancer cells and adipocytes in peritumoral breast AT has a crucial role in the progression and metastasis of breast cancer [12,13,28,29]. Breast cancer cells induce dedifferentiation of peritumoral adipocytes, the so-called cancer-associated adipocytes, which show a decreased adipocyte markers expression and an increased proinflammatory cytokines expression [13,28,29]. Furthermore, cancer cells also induce the release of intracellular free fatty acids from cancer-associated adipocytes, which not only provide energy to cancer cells, but also induce metabolic remodeling of cancer cells [13,29,30]. Conversely, cancer-associated adipocytes promote growth, proliferation, invasion, and metastasis of breast cancer cells by excreting proinflammatory and protumor adipokines, inducing extracellular matrix remodeling and releasing various metabolic substrates [12,13,29]. 

In the previous studies, FDG uptake of AT in the PET/CT images was used as an imaging parameter reflecting the qualitative changes of the AT microenvironment induced by interaction between cancer cells and adipocytes [19,31]. In patients with pancreatic cancer, gastric cancer, and prostate cancer, the mean FDG uptake of peritumoral AT showed a significant positive relationship with tumor aggressiveness and was an independent predictor of survival [18,19,20]. The results of this study demonstrated significant differences in textural features of FDG PET/CT between peritumoral and contralateral breast AT and most of the textural parameters of peritumoral AT, including mean FDG uptake, also showed significant differences between the patients with and without axillary lymph node metastasis. Furthermore, the textural features of peritumoral AT showed significant correlation with size and molecular subtypes of primary breast cancers. These findings suggest that the FDG PET/CT imaging features of peritumoral breast AT were distinct from those of contralateral breast AT and were significantly correlated with the characteristics of breast cancer and presence of axillary lymph node metastasis. There are several possible explanations for this relationship. In a previous study of tumor and peritumoral AT tissues in breast cancer patients, the increased expression of hexokinase 2 and glucose-6-phosphate dehydrogenase was observed not only in cancer cells, but also in peritumoral adipocytes, suggesting that breast cancer promoted the Warburg effect in cancer-associated adipocytes [14]. Because more prominent expression of hexokinase 2 was observed in adipocytes at the invasive front of cancer than those at the distant site [14], this reprogramming of glucose metabolism in adipocytes could increase the FDG uptake heterogeneity and intensity in peritumoral breast AT. Furthermore, cancer-associated adipocytes recruit macrophages in peritumoral AT via secreting diverse kinds of proinflammatory cytokines with significant interaction [32,33]. This interaction is known to increase the inflammatory response in peritumoral AT and facilitate invasion and axillary lymph node metastasis of breast cancer cells [34,35]. Since the macrophage-induced inflammatory response was suggested as a cause of increased FDG uptake in AT in previous studies [19,31], the PET/CT imaging features of peritumoral breast AT with severe macrophage infiltration differed from those of normal breast AT. Another possible explanation for the relationship is cancer cell infiltration into peritumoral AT, which could increase FDG uptake intensity and heterogeneity of peritumoral breast AT and the risk of axillary node metastasis [36,37]. 

Currently, visual analysis and LN SUVmax are routinely used PET/CT features in diagnosing axillary lymph node metastasis of breast cancer [9]. However, most previous studies showed a sensitivity <60% for detecting axillary node metastasis with these methods, and therefore several attempts have been made to improve the diagnostic ability of FDG PET/CT, such as using dual-phase PET/CT or textural features of the primary tumor [4,8,9,11]. In our study, we investigated the clinical significance of textural features of peritumoral breast AT to predict axillary lymph node metastasis and demonstrated their utility in diagnosing lymph node metastasis. Among the textural features of peritumoral breast AT, GLCM entropy showed the maximum diagnostic ability for predicting axillary lymph node metastasis of breast cancer, showing significantly higher diagnostic ability than visual analysis, and was comparable to LN SUVmax. GLCM is the probability of observing a pair of values in image voxels at a given distance in a given direction, and entropy measures the randomness of distribution of voxel pairs [38]. Therefore, the GLCM entropy of a lesion represents intra-lesion metabolic heterogeneity [38,39]. GLCM entropy extracted from FDG PET/CT images showed high robustness independent of the reconstruction algorithm, noise, iteration number, and matrix size and is regarded as one of the feasible textural features that can be used even in multicenter trials [40]. In this study, the patients with axillary lymph node metastasis showed increased GLCM entropy in peritumoral breast AT, and GLCM entropy showed high sensitivity and NPV for predicting axillary lymph node metastasis. Furthermore, using both PET/CT findings of axillary lymph nodes and GLCM entropy of peritumoral breast AT, the predictive value for axillary lymph node metastases was further enhanced. Because over 95% of the patients with positive axillary lymph node findings and high GLCM entropy value had axillary lymph node metastasis, axillary lymph node dissection could be recommended for these patients. Textural features of peritumoral breast AT such as GLCM entropy might help decide treatment strategies for patients with breast cancer. 

Interestingly, significant differences in GLCM entropy as well as SUV histogram entropy were observed based on the results of the visual analysis of FDG PET/CT. The highest value was found in the patients with true-positive findings, followed by the patients with false-negative readings and subsequently, the patients with false-positive and true-negative results. A false-negative result of axillary lymph node metastasis on PET/CT was significantly correlated with small tumor size and micrometastasis; therefore, PET/CT findings of axillary lymph nodes are thought to be associated with metastatic lesion burden [9,41,42]. In this regard, GLCM entropy and SUV histogram entropy of peritumoral breast AT are supposed to reflect the metastatic lesion burden, indicating that the GLCM entropy and SUV histogram entropy increase with the increase in metastatic lesion burden. Further, because the patients with false-negative findings showed significantly higher values of GLCM entropy and SUV histogram entropy than those without axillary lymph node metastasis, both features still demonstrated a high AUC value with high sensitivity and NPV for predicting axillary lymph node metastases in the subgroup analysis of the patients who showed negative findings for axillary lymph nodes involvement in the PET/CT images. Our results suggest that textural features of peritumoral breast AT such as GLCM entropy and SUV histogram entropy might be used as alternative imaging parameters for predicting axillary lymph node metastasis in patients with negative PET/CT findings. Although the clinical significance of textural features of peritumoral breast AT alone could be limited, combining these features with the primary tumor findings on various imaging examinations might provide useful information in stratifying the risk of axillary lymph node metastasis. 

The underlying mechanism of relationship between GLCM entropy and axillary nodal metastasis is not clearly known. The possible explanation is that the metabolic reprogramming of adipocytes and macrophage-induced inflammatory response have shown heterogeneous appearance in peritumoral breast AT [14,43], which might be reflected by GLCM entropy. Considering that both GLCM entropy and SUV histogram entropy showed high diagnostic ability with AUC values >0.800, the degree of heterogeneous distribution of cancer-associated adipocytes and macrophage infiltration in peritumoral AT might have a significant relationship with node metastasis. However, because several causative factors other than metabolic heterogeneity of cells in breast AT could affect GLCM entropy, further studies are necessary to explain the precise underlying mechanism. 

There are several limitations in this study. First, this was a retrospective study performed at a single medical center, suggesting an inherent risk of selection bias. Further external validation of our results in a large pooled population is warranted. Second, although previous studies also used a 1 cm distance to the tumor margin for defining peritumoral AT, the proper measurement of imaging features of peritumoral AT is yet to be established [15,17], which could limit its clinical application. Further study that compares the use of various distances in defining peritumoral breast AT would be helpful. Finally, because of the retrospective nature of the study, histopathological analysis of peritumoral breast AT was not performed to identify the precise mechanisms involved.

## 5. Conclusions

In this study, there were significant differences in the FDG PET/CT textural features between peritumoral and contralateral breast AT, and the diagnostic potential of the peritumoral AT textural features was demonstrated in predicting axillary lymph node metastasis of patients with breast cancer. Among the textural features, GLCM entropy of peritumoral breast AT showed the highest diagnostic ability with high sensitivity and NPV, which was comparable to LN SUVmax. In the subgroup analysis of the patients with negative findings of axillary lymph nodes on PET/CT, GLCM entropy still showed a high diagnostic ability for predicting axillary lymph node metastasis. Textural features of peritumoral breast AT on FDG PET/CT might be used to assess the risk of axillary lymph node metastasis in patients with breast cancer.

## Figures and Tables

**Table 1:** Baseline characteristics of the 326 patients.

Characteristics		Number of Patients (%)
Age (years)		52 (30–85) ^1^
Obesity	Underweight/normal	130 (39.9%)
	Overweight/obesity	196 (60.1%)
Menopausal status	Premenopausal	135 (41.4%)
	Postmenopausal	191 (58.6%)
Histopathology	Intraductal carcinoma	287 (88.0%)
	Intralobular carcinoma	39 (12.0%)
Histologic grade	Grade 1	80 (24.6%)
	Grade 2	160 (49.2%)
	Grade 3	85 (26.2%)
Tumor size (cm)		2.0 (0.4–15.0) ^1^
T stage	T1–T2	295 (90.5%)
	T3–T4	31 (9.5%)
Lymph node metastasis	No	207 (63.5%)
	Yes	119 (36.5%)
Molecular subtypes	Luminal A	64 (19.6%)
	Luminal B	182 (55.8%)
	HER2-enriched (non-luminal)	42 (12.9%)
	Triple-negative	38 (11.7%)
Maximum SUV of the primary tumor		3.96 (1.50–37.90) ^1^
Maximum SUV of the axillary lymph node		1.08 (0.36–27.07) ^1^
Neoadjuvant chemotherapy	No	296 (90.8%)
	Yes	30 (9.2%)
Treatment for primary breast cancer	Breast-conserving surgery	229 (70.2%)
	Total mastectomy	97 (29.8%)
Treatment for axillary lymph nodes	Sentinel lymph node biopsy	184 (56.4%)
	Axillary lymph node dissection	142 (43.6%)
Adjuvant treatment	No	5 (1.5%)
	CTx + RTx_HTx	159 (48.8%)
	RTx + HTx	94 (28.8%)
	HTx	29 (8.9%)
	CTx	16 (4.9%)
	CTx + HTx	16 (4.9%)
	CTx + RTx	4 (1.2%)
	RTx	3 (0.9%)

^1^ Median value (range). Maximum SUV of the axillary lymph node was measured in 278 patients. CTx, chemotherapy; HER2, human epidermal growth factor receptor 2; HTx, hormone therapy; RTx, radiotherapy; SUV, standardized uptake value.

**Table 2:** Diagnostic ability of the visual analysis and textural features of FDG PET/CT with AUC values > 0.750 for predicting axillary lymph node metastasis.

Parameters	Cutoff value	AUC (95% CI)	Sensitivity (%)	Specificity (%)	PPV (%)	NPV (%)
Axillary lymph node parameters						
Visual analysis	-	0.739 (0.689–0.785)	53.8	93.7	83.1	77.9
Maximum SUV of the axillary lymph node ^1^	1.58	0.793 (0.741–0.839)	56.9	91.4	82.5	74.7
Peritumoral breast adipose tissue textural parameters						
GLCM entropy	3.16	0.830 (0.784–0.869)	82.4	74.4	64.9	88.0
SUV histogram entropy	1.90	0.815 (0.768–0.855)	84.0	70.5	62.1	88.5
GLCM contrast	0.63	0.780 (0.731–0.823)	84.0	64.3	57.5	87.5
GLZLM zone-length nonuniformity	7.27	0.766 (0.716–0.811)	58.8	83.1	66.7	77.8
GLZLM high grey-level zone emphasis	21.31	0.765 (0.715–0.810)	67.2	74.9	60.6	79.9
GLRLM high grey-level run emphasis	9.41	0.763 (0.713–0.808)	75.6	66.7	56.6	82.6
GLRLM short-run high grey-level emphasis	7.25	0.761 (0.711–0.806)	79.8	62.8	55.2	84.4

^1^ Calculated in the 278 patients who featured axillary lymph nodes in the PET/CT images. AUC, area under the receiver operating characteristic curve; CI, confidence interval; GLCM, grey-level co-occurrence matrix; GLRLM, grey-level run-length matrix; GLZLM, grey-level zone-length matrix; NPV, negative predictive value; PPV, positive predictive value; SUV, standardized uptake value.

**Table 3:** Rates of the patients with axillary lymph node metastasis based on the combination of axillary lymph node findings on PET/CT (visual analysis and maximum SUV) with GLCM entropy and SUV histogram entropy of peritumoral breast adipose tissue.

		Visual Analysis		Maximum SUV of the Axillary Lymph Node ^1^	
		Negative	Positive	≤1.58	>1.58
GLCM entropy	≤3.16	15/158 (9.5%)	6/16 (37.5%)	13/122 (10.7%)	6/17 (35.3%)
	>3.16	40/91 (44.0%)	58/61 (95.1%)	37/76 (48.7%)	60/63 (95.2%)
SUV histogram entropy	≤1.90	16/152 (10.5%)	3/12 (25.0%)	14/118 (11.9%)	3/14 (21.4%)
	>1.90	39/97 (40.2%)	61/65 (93.8%)	36/80 (45.0%)	63/66 (95.5%)

^1^ Assessed in the 278 patients who showed measurable axillary lymph nodes in the PET/CT images. GLCM, grey-level co-occurrence matrix; SUV, standardized uptake value.

## Data Availability

The datasets generated during and/or analyzed during this study are available from the corresponding authors upon reasonable request.

## Supplementary Materials

The following are available online at https://www.mdpi.com/article/10.3390/jpm11101029/s1, Table S1: List of the 38 textural features measured in peritumoral and contralateral breast adipose tissue, Table S2: Pairwise comparison of the 38 textural features between peritumoral and contralateral breast adipose tissue, Table S3: Comparison of the 38 textural features of peritumoral breast adipose tissue between the patients with and without axillary lymph node metastasis, Table S4. Correlation of the primary tumor size with the 38 textural features of peritumoral breast adipose tissue, Table S5. Correlation of molecular subtypes of the primary tumors with the 38 textural features of peritumoral breast adipose tissue, Table S6. Diagnostic ability of the primary tumor parameters and textural features of FDG PET/CT with AUC values < 0.750 for predicting axillary lymph node metastasis.
