# Supplementary material for: Clinical Significance of Peritumoral Adipose Tissue PET/CT Imaging Features for Predicting Axillary Lymph Node Metastasis in Patients with Breast Cancer
Source: J Pers Med. 2021 Oct 15;11(10):1029. doi: 10.3390/jpm11101029 (PMC8540268; doi:10.3390/jpm11101029)
Supplement: Supplementary file 1 [file jpm-11-01029-s001.zip › jpm-1368084-supplementary.pdf]

Table S1. List of the 38 textural features measured in peritumoral and contralateral breast adipose tissue.

Order	Matrix	Index
First-order		SUV mean
		SUV median
		SUV standard deviation
		SUV skewness
		SUV kurtosis
		SUV histogram entropy
		SUV histogram energy
Higher-order	Grey-level co-occurrence matrix	Contrast
		Correlation
		Dissimilarity
		Energy
		Entropy
		Homogeneity
	Neighborhood grey-level different matrix	Busyness
		Coarseness
		Contrast
	Grey-level run-length matrix	Grey-level non-uniformity for run
		High grey-level run emphasis
		Long-run emphasis
		Long-run high grey-level emphasis
		Long-run low grey-level emphasis
		Low grey-level run emphasis
		Run length non-uniformity
		Run percentage
		Short-run emphasis
		Short-run high grey-level emphasis
		Short-run low grey-level emphasis
	Grey-level zone-length matrix	Grey-level non-uniformity for zone
		High grey-level zone emphasis
		Long-zone emphasis
		Long-zone high grey-level emphasis
		Long-zone low grey-level emphasis
		Low grey-level zone emphasis
		Short-zone emphasis
		Short-zone high grey-level emphasis
		Short-zone low grey-level emphasis
		Zone length non-uniformity
		Zone percentage

SUV, standardized uptake value

Table S2. Pairwise comparison of the 38 textural features between peritumoral and contralateral breast adipose tissue.

Imaging feature	Peritumoral breast adipose tissue	Contralateral breast adipose tissue	P-value
SUV mean	0.71 ± 0.22	0.39 ± 0.11	<0.001
SUV median	0.62 ± 0.19	0.35 ± 0.11	<0.001
SUV standard deviation	0.36 ± 0.24	0.16 ± 0.06	<0.001
SUV skewness	1.32 ± 0.71	1.11 ± 0.54	<0.001
SUV kurtosis	5.72 ± 3.81	4.55 ± 1.95	<0.001
SUV histogram entropy	1.91 ± 0.53	1.21 ± 0.37	<0.001
SUV histogram energy	0.35 ± 0.12	0.51 ± 0.14	<0.001
GLCM contrast	1.16 ± 1.61	0.22 ± 0.10	<0.001
GLCM correlation	0.59 ± 0.13	0.64 ± 0.15	<0.001
GLCM dissimilarity	0.60 ± 0.36	0.22 ± 0.09	<0.001
GLCM energy	0.21 ± 0.12	0.40 ± 0.16	<0.001
GLCM entropy	3.15 ± 0.91	1.87 ± 0.57	<0.001
GLCM homogeneity	0.76 ± 0.09	0.89 ± 0.04	<0.001
NGLDM busyness	3.77 ± 3.87	84.48 ± 517.52	0.005
NGLDM coarseness	0.03 ± 0.02	0.01 ± 0.02	<0.001
NGLDM contrast	0.03 ± 0.01	0.02 ± 0.01	<0.001
GLRLM grey-level non-uniformity for run	115.51 ± 108.09	359.47 ± 288.24	<0.001
GLRLM high grey-level run emphasis	11.55 ± 9.45	4.15 ± 1.62	<0.001
GLRLM long-run emphasis	3.98 ± 1.76	14.78 ± 11.49	<0.001
GLRLM long-run high grey-level emphasis	28.12 ± 12.10	35.84 ± 15.12	<0.001
GLRLM long-run low grey-level emphasis	1.20 ± 1.19	10.41 ± 11.90	<0.001
GLRLM low grey-level run emphasis	0.24 ± 0.12	0.49 ± 0.16	<0.001
GLRLM run length non-uniformity	222.95 ± 211.27	216.51 ± 166.39	0.643
GLRLM run percentage	0.66 ± 0.11	0.40 ± 0.11	<0.001
GLRLM short-run emphasis	0.72 ± 0.11	0.46 ± 0.11	<0.001
GLRLM short-run high grey-level emphasis	9.99 ± 10.00	2.40 ± 1.32	<0.001
GLRLM short-run low grey-level emphasis	0.16 ± 0.06	0.18 ± 0.05	<0.001
GLZLM grey-level non-uniformity for zone	5.42 ± 4.60	4.24 ± 2.87	<0.001
GLZLM high grey-level zone emphasis	26.14 ± 27.30	7.83 ± 3.83	<0.001
GLZLM long-zone emphasis	10335.97 ± 26150.45	662082.99 ± 1613753.04	<0.001
GLZLM long-zone high grey-level emphasis	49437.46 ± 120535.44	1118854.51 ± 2140316.99	<0.001
GLZLM long-zone low grey-level emphasis	3345.71 ± 9415.51	556238.77 ± 153769.33	<0.001
GLZLM low grey-level zone emphasis	0.19 ± 0.13	0.35 ± 0.15	<0.001
GLZLM short-zone emphasis	0.39 ± 0.16	0.25 ± 0.14	<0.001
GLZLM short-zone high grey-level emphasis	15.09 ± 22.31	2.50 ± 2.10	<0.001
GLZLM short-zone low grey-level emphasis	0.05 ± 0.05	0.08 ± 0.09	<0.001
GLZLM zone length non-uniformity	8.42 ± 12.46	2.10 ± 1.91	<0.001
GLZLM zone percentage	0.06 ± 0.05	0.01 ± 0.01	<0.001

All data are expressed in mean ± standard deviation

GLCM, grey-level co-occurrence matrix; GLRLM, grey-level run-length matrix; GLZLM, grey-level zone-length matrix; NGLDM, neighborhood grey-level different matrix; SUV, standardized uptake value

Table S3. Comparison of the 38 textural features of peritumoral breast adipose tissue between the patients with and without axillary lymph node metastasis.

Imaging feature	Patients with axillary lymph node metastasis (n=119)	Patients with no axillary lymph node metastasis (n=207)	P-value
SUV mean	0.83 ± 0.24	0.64 ± 0.18	<0.001
SUV median	0.70 ± 0.20	0.57 ± 0.16	<0.001
SUV standard deviation	0.45 ± 0.24	0.31 ± 0.22	<0.001
SUV skewness	1.55 ± 0.76	1.20 ± 0.66	<0.001
SUV kurtosis	6.86 ± 4.97	5.07 ± 2.76	<0.001
SUV histogram entropy	2.27 ± 0.48	1.71 ± 0.44	<0.001
SUV histogram energy	0.30 ± 0.10	0.38 ± 0.12	<0.001
GLCM contrast	1.83 ± 2.19	0.77 ± 0.97	<0.001
GLCM correlation	0.59 ± 0.12	0.59 ± 0.14	0.508
GLCM dissimilarity	0.76 ± 0.40	0.51 ± 0.31	<0.001
GLCM energy	0.16 ± 0.10	0.24 ± 0.13	<0.001
GLCM entropy	3.78 ± 0.79	2.79 ± 0.77	<0.001
GLCM homogeneity	0.72 ± 0.09	0.78 ± 0.08	<0.001
NGLDM busyness	2.77 ± 3.31	4.35 ± 4.06	<0.001
NGLDM coarseness	0.02 ± 0.02	0.03 ± 0.02	0.028
NGLDM contrast	0.03 ± 0.02	0.02 ± 0.01	0.001
GLRLM grey-level non-uniformity for run	131.89 ± 153.92	106.10 ± 67.95	0.085
GLRLM high grey-level run emphasis	15.22 ± 9.71	9.45 ± 8.64	<0.001
GLRLM long-run emphasis	3.35 ± 1.43	4.34 ± 1.83	<0.001
GLRLM long-run high grey-level emphasis	30.93 ± 12.88	26.50 ± 11.35	0.002
GLRLM long-run low grey-level emphasis	0.81 ± 0.68	1.43 ± 1.35	<0.001
GLRLM low grey-level run emphasis	0.19 ± 0.10	0.27 ± 0.12	<0.001
GLRLM run length non-uniformity	235.62 ± 272.36	211.15 ± 148.83	0.065
GLRLM run percentage	0.70 ± 0.09	0.63 ± 0.11	<0.001
GLRLM short-run emphasis	0.77 ± 0.08	0.69 ± 0.11	<0.001
GLRLM short-run high grey-level emphasis	13.37 ± 9.62	8.05 ± 9.72	<0.001
GLRLM short-run low grey-level emphasis	0.14 ± 0.06	0.17 ± 0.06	<0.001
GLZLM grey-level non-uniformity for zone	7.42 ± 5.83	4.27 ± 3.22	<0.001
GLZLM high grey-level zone emphasis	35.46 ± 25.27	20.78 ± 27.03	<0.001
GLZLM long-zone emphasis	6961.32 ± 24194.57	12275.98 ± 27077.84	0.069
GLZLM long-zone high grey-level emphasis	40100.36 ± 118258.97	54805.16 ± 121784.21	0.286
GLZLM long-zone low grey-level emphasis	1749.50 ± 7270.90	4263.34 ± 10355.35	0.011
GLZLM low grey-level zone emphasis	0.13 ± 0.09	0.22 ± 0.14	<0.001
GLZLM short-zone emphasis	0.45 ± 0.13	0.36 ± 0.16	<0.001
GLZLM short-zone high grey-level emphasis	21.65 ± 20.77	11.33 ± 22.34	<0.001
GLZLM short-zone low grey-level emphasis	0.04 ± 0.04	0.06 ± 0.06	0.004
GLZLM zone length non-uniformity	14.69 ± 17.27	4.82 ± 6.19	<0.001
GLZLM zone percentage	0.08 ± 0.05	0.04 ± 0.04	<0.001

All data are expressed in mean ± standard deviation

GLCM, grey-level co-occurrence matrix; GLRLM, grey-level run-length matrix; GLZLM, grey-level zone-length

matrix; NGLDM, neighborhood grey-level different matrix; SUV, standardized uptake value

Table S4. Correlation of the primary tumor size with the 38 textural features of peritumoral breast adipose tissue.

Imaging features	P-value	Correlation coefficient
SUV mean	<0.001	0.390
SUV median	<0.001	0.333
SUV standard deviation	<0.001	0.424
SUV skewness	0.010	0.180
SUV kurtosis	0.010	0.181
SUV histogram entropy	<0.001	0.383
SUV histogram energy	<0.001	-0.399
GLCM contrast	<0.001	0.379
GLCM correlation	0.177	0.098
GLCM dissimilarity	<0.001	0.363
GLCM energy	<0.001	-0.359
GLCM entropy	<0.001	0.352
GLCM homogeneity	<0.001	-0.339
NGLDM busyness	0.063	-0.103
NGLDM coarseness	<0.001	-0.402
NGLDM contrast	0.016	0.133
GLRLM grey-level non-uniformity for run	0.055	0.117
GLRLM high grey-level run emphasis	<0.001	0.423
GLRLM long-run emphasis	<0.001	-0.248
GLRLM long-run high grey-level emphasis	<0.001	0.274
GLRLM long-run low grey-level emphasis	<0.001	-0.266
GLRLM low grey-level run emphasis	<0.001	-0.322
GLRLM run length non-uniformity	0.114	0.101
GLRLM run percentage	<0.001	0.281
GLRLM short-run emphasis	<0.001	0.346
GLRLM short-run high grey-level emphasis	<0.001	0.440
GLRLM short-run low grey-level emphasis	<0.001	-0.306
GLZLM grey-level non-uniformity for zone	<0.001	0.549
GLZLM high grey-level zone emphasis	<0.001	0.457
GLZLM long-zone emphasis	0.030	-0.164
GLZLM long-zone high grey-level emphasis	0.087	-0.095
GLZLM long-zone low grey-level emphasis	<0.001	-0.195
GLZLM low grey-level zone emphasis	<0.001	-0.434
GLZLM short-zone emphasis	<0.001	0.387
GLZLM short-zone high grey-level emphasis	<0.001	0.451
GLZLM short-zone low grey-level emphasis	0.318	-0.055
GLZLM zone length non-uniformity	<0.001	0.501
GLZLM zone percentage	<0.001	0.348

GLCM, grey-level co-occurrence matrix; GLRLM, grey-level run-length matrix; GLZLM, grey-level zone-length matrix; NGLDM, neighborhood grey-level different matrix; SUV, standardized uptake value

Table S5. Correlation of molecular subtypes of the primary tumors with the 38 textural features of peritumoral breast adipose tissue.

Imaging features	P-value	Luminal A	Luminal B	HER2-enriched (non-luminal)	Triple negative
SUV mean	<0.001①	0.62 (0.52–0.76)	0.65 (0.54–0.79)	0.79 (0.65–0.95)	0.77 (0.65–0.92)
SUV median	<0.001①	0.53 (0.44–0.68)	0.56 (0.46–0.70)	0.69 (0.57–0.82)	0.66 (0.55–0.80)
SUV standard deviation	<0.001②	0.28 (0.20–0.34)	0.28 (0.22–0.39)	0.38 (0.25–0.52)	0.39 (0.32–0.55)
SUV skewness	0.004③	1.22 (0.76–1.67)	1.20 (0.83–1.65)	1.25 (0.87–1.84)	1.58 (1.16–2.12)
SUV kurtosis	0.002③	4.41 (3.13–6.30)	4.35 (3.47–6.16)	5.07 (3.69–7.29)	6.27 (4.34–9.20)
SUV histogram entropy	<0.001②	1.72 (1.45–2.05)	1.86 (1.59–2.17)	2.09 (1.66–2.41)	2.05 (1.81–2.54)
SUV histogram energy	<0.001②	0.36 (0.29–0.45)	0.34 (0.28–0.41)	0.29 (0.22–0.38)	0.29 (0.22–0.33)
GLCM contrast	<0.001②	0.53 (0.33–0.83)	0.68 (0.39–1.04)	0.86 (0.47–1.92)	1.03 (0.69–1.98)
GLCM correlation	0.432	0.64 (0.54–0.69)	0.59 (0.49–0.68)	0.62 (0.47–0.69)	0.62 (0.53–0.69)
GLCM dissimilarity	<0.001②	0.43 (0.31–0.61)	0.52 (0.35–0.65)	0.58 (0.39–0.92)	0.65 (0.51–0.86)
GLCM energy	<0.001③	0.20 (0.15–0.31)	0.19 (0.14–0.26)	0.17 (0.09–0.23)	0.15 (0.09–0.19)
GLCM entropy	0.001③	2.83 (2.35–3.48)	2.96 (2.56–3.62)	3.21 (2.70–4.34)	3.38 (2.88–4.32)
GLCM homogeneity	<0.001②	0.80 (0.74–0.85)	0.77 (0.72–0.83)	0.74 (0.65–0.81)	0.72 (0.68–0.79)
NGLDM busyness	0.124	2.89 (1.86–4.78)	2.75 (1.85–4.31)	2.52 (1.06–4.57)	1.86 (1.15–3.95)
NGLDM coarseness	0.002③	0.02 (0.02–0.03)	0.02 (0.01–0.03)	0.02 (0.01–0.03)	0.01 (0.01–0.02)
NGLDM contrast	0.415	0.02 (0.01–0.03)	0.02 (0.02–0.03)	0.02 (0.01–0.03)	0.02 (0.02–0.03)
GLRLM grey-level non-uniformity for run	0.287	81.78 (57.75–121.89)	90.98 (64.86–125.17)	87.03 (64.79–166.56)	104.79 (72.11–148.20)
GLRLM high grey-level run emphasis	<0.001①	8.04 (5.79–10.74)	9.08 (6.47–12.70)	12.43 (7.82–18.15)	12.49 (9.12–18.57)
GLRLM long-run emphasis	0.008④	4.02 (2.75–5.70)	3.46 (2.82–4.84)	3.11 (2.34–4.37)	2.97 (2.43–3.85)
GLRLM long-run high grey-level emphasis	0.367	26.17 (21.51–0.42)	26.22 (20.64–30.47)	28.07 (22.97–38.32)	27.20 (21.91–33.85)
GLRLM long-run low grey-level emphasis	0.007⑤	1.15 (0.53–1.96)	0.85 (0.50–1.50)	0.60 (0.38–1.15)	0.76 (0.42–1.06)
GLRLM low grey-level run emphasis	<0.001⑥	0.26 (0.17–0.33)	0.22 (0.15–0.33)	0.18 (0.12–0.21)	0.19 (0.12–0.28)
GLRLM run length non-uniformity	<0.001①	128.33 (89.06–197.60)	151.18 (100.36–246.12)	220.22 (154.72–363.45)	259.83 (178.87–417.47)
GLRLM run percentage	0.006⑦	0.63 (0.53–.72)	0.67 (0.58–0.72)	0.71 (0.61–0.78)	0.71 (0.63–0.77)
GLRLM short-run emphasis	<0.001⑦	0.70 (0.60–0.78)	0.74 (0.64–0.79)	0.79 (0.69–0.84)	0.79 (0.71–0.82)
GLRLM short-run high grey-level emphasis	<0.001①	6.23 (3.90–9.06)	7.37 (4.77–11.05)	11.22 (6.33–15.28)	10.64 (7.48–17.09)
GLRLM short-run low grey-level emphasis	<0.001⑥	0.16 (0.13–0.21)	0.16 (0.11–0.21)	0.12 (0.09–0.15)	0.13 (0.09–0.20)
GLZLM grey-level non-uniformity for zone	<0.001⑧	2.89 (1.93–4.33)	4.05 (2.60–5.85)	6.32 (3.32–8.91)	6.77 (4.26–12.88)
GLZLM high grey-level zone emphasis	<0.001①	15.19 (9.45–23.29)	17.22 (11.67–27.52)	27.80 (14.36–49.95)	27.55 (21.29–43.98)
GLZLM long-zone emphasis	0.019④	4428.92 (1628.29–14948.90)	3423.44 (1675.49–7196.58)	2651.08 (934.901–6236.75)	1820.45 (923.71–4305.92)
GLZLM long-zone high grey-level emphasis	0.068	24335.86 (11644.71–62788.31)	19646.19 (10254.94–41243.34)	16286.62 (6725.68–48634.24)	12087.05 (6839.86–0189.84)
GLZLM long-zone low grey-level emphasis	0.141	1067.27 (343.51–4951.36)	820.83 (291.52–2347.24)	407.13 (161.31–1283.50)	403.23 (174.91–521.02)

GLZLM low grey-level zone emphasis	<0.001①	0.19 (0.12–0.28)	0.16 (0.10–0.26)	0.11 (0.06–0.22)	0.09 (0.06–0.14)
GLZLM short-zone emphasis	<0.001⑧	0.31 (0.17–0.44)	0.40 (0.30–0.49)	0.51 (0.41–0.58)	0.52 (0.39–0.56)
GLZLM short-zone high grey-level emphasis	<0.001①	5.30 (2.21–11.43)	7.95 (3.86–14.37)	15.27 (6.65–39.25)	18.01 (11.56–8.37)
GLZLM short-zone low grey-level emphasis	0.062	0.03 (0.01–0.05)	0.03 (0.02–0.06)	0.03 (0.02–0.08)	0.02 (0.01–0.04)
GLZLM zone length non-uniformity	<0.001⑧	1.92 (1.22–6.97)	3.39 (1.80–8.38)	7.54 (2.54–15.69)	9.13 (4.87–19.49)
GLZLM zone percentage	<0.001②	0.03 (0.02–0.05)	0.04 (0.03–0.06)	0.06 (0.03–0.10)	0.8 (0.05–0.10)

GLCM, grey-level co-occurrence matrix; GLRLM, grey-level run-length matrix; GLZLM, grey-level zone-length matrix; NGLDM, neighborhood grey-level different matrix; SUV, standardized uptake value

① On post-hoc analysis, patients with HER2-enriched and triple negative breast cancers showed significantly different values from those with luminal A and B breast cancers ($p<0.05$).

② On post-hoc analysis, patients with triple negative breast cancer showed significantly different values from those with luminal A and B breast cancers ($p<0.05$), and patients with HER2-enriched breast cancer showed significantly different values from those with luminal A breast cancer ($p<0.05$). .

③ On post-hoc analysis, patients with triple negative breast cancer showed significantly different values from those with luminal A and B breast cancers ($p<0.05$).

④ On post-hoc analysis, patients with triple negative breast cancer showed significantly different values from those with luminal A breast cancer ($p<0.05$).

⑤ On post-hoc analysis, patients with HER2-enriched breast cancer showed significantly different values from those with luminal A breast cancer ($p<0.05$).

⑥ On post-hoc analysis, patients with HER2-enriched breast cancer showed significantly different values from those with luminal A and B breast cancers ($p<0.05$).

⑦ On post-hoc analysis, patients with HER2-enriched and triple negative breast cancers showed significantly different values from those with luminal A breast cancer ($p<0.05$).

⑧ On post-hoc analysis, patients with HER2-enriched and triple negative breast cancers showed significantly different values from those with luminal A and B breast cancers ($p<0.05$), and patients with luminal B breast cancer showed significantly different values from those with luminal A breast cancer ($p<0.05$).

Table S6. Diagnostic ability of the primary tumor parameters and textural features of FDG PET/CT with AUC values <0.750 for predicting axillary lymph node metastasis

Parameters	Cut-off value	AUC (95% CI)	Sensitivity (%)	Specificity (%)	PPV (%)	NPV (%)
Primary tumor parameters						
Tumor size	1.7	0.693 (0.640–0.742)	76.5	50.2	46.9	78.8
Maximum SUV of primary tumor	3.38	0.750 (0.699–0.796)	83.2	59.4	54.1	86.0
Peritumoral breast adipose tissue textural parameters						
SUV mean	0.72	0.747 (0.696–0.793)	68.9	72.5	59.0	80.2
SUV median	0.54	0.702 (0.649–0.751)	80.7	47.8	47.1	81.1
SUV standard deviation	0.33	0.747 (0.697–0.794)	69.9	73.0	59.4	80.3
SUV skewness	1.36	0.634 (0.580–0.687)	58.0	66.7	50.0	73.4
SUV kurtosis	4.97	0.639 (0.584–0.691)	60.5	62.8	48.3	73.4
SUV histogram energy	0.34	0.715 (0.663–0.764)	77.3	58.0	51.4	81.6
GLCM dissimilarity	0.51	0.740 (0.689–0.787)	77.3	63.8	55.1	83.0
GLCM energy	0.21	0.714 (0.662–0.763)	83.2	52.7	50.3	84.5
GLCM homogeneity	0.78	0.724 (0.672–0.772)	78.2	59.9	52.8	82.7
NGLDM busyness	1.76	0.654 (0.599–0.705)	42.0	81.6	56.8	71.0
NGLDM coarseness	0.02	0.594 (0.538–0.648)	71.4	48.3	44.3	74.6
NGLDM contrast	0.03	0.586 (0.530–0.640)	52.1	68.6	48.8	71.4
GLRLM long-run emphasis	3.46	0.677 (0.624–0.728)	69.7	61.4	50.9	77.9
GLRLM long-run high grey-level emphasis	24.14	0.620 (0.565–0.673)	72.3	48.8	44.8	75.4
GLRLM long-run low grey-level emphasis	1.30	0.678 (0.625–0.729)	89.1	42.5	47.1	87.1
GLRLM low grey-level run emphasis	0.20	0.694 (0.641–0.744)	67.2	63.3	51.1	77.1
GLRLM run percentage	0.65	0.689 (0.635–0.739)	74.8	57.0	50.0	79.7
GLRLM short-run emphasis	0.73	0.703 (0.657–0.755)	74.8	59.9	51.7	80.5
GLRLM short-run low grey-level emphasis	0.15	0.672 (0.618–0.723)	67.2	61.8	50.3	76.6
GLZLM grey-level non-uniformity for zone	5.19	0.703 (0.650–0.752)	58.8	75.8	58.3	76.2
GLZLM long-zone low grey-level emphasis	925.03	0.671 (0.617–0.721)	74.8	52.2	47.3	78.3
GLZLM low grey-level zone emphasis	0.17	0.731 (0.685–0.781)	79.0	57.0	51.4	82.5
GLZLM short-zone emphasis	0.39	0.660 (0.606–0.711)	71.4	56.0	48.3	77.3
GLZLM short-zone high grey-level emphasis	12.74	0.748 (0.697–0.794)	62.2	78.7	62.7	78.4
GLZLM short-zone low grey-level emphasis	0.03	0.558 (0.502–0.613)	52.9	60.4	43.4	69.1
GLZLM zone percentage	0.03	0.722 (0.670–0.770)	93.3	41.6	47.8	91.5

AUC, area under the receiver operating characteristic curve; CI, confidence interval; GLCM, grey-level co-occurrence matrix; GLRLM, grey-level run-length matrix; GLZLM, grey-level zone-length matrix; NGLDM, neighborhood grey-level different matrix; NPV, negative predictive value; PPV, positive predictive value; SUV, standardized uptake value
